# Spatial quantile regression with application to high and low child birth weight in Malawi

**DOI:** 10.1186/s12889-019-7949-9

**Published:** 2019-11-29

**Authors:** Alfred Ngwira

**Affiliations:** Department of Basic Sciences, Lilongwe University of Agriculture and Natural Resources, Lilongwe, Malawi

**Keywords:** Spatial, Quantile, Bayesian, INLA

## Abstract

**Background:**

Child low and high birth weight are important public health problems. Many studies have looked at factors of low and high birth weight using mean regression. This study aimed at using quantile regression to find out determinants of low and high birth weight.

**Methods:**

Spatial quantile regression models at 0.05 and 0.95 percentiles of birth weight were fitted to 13,087 children birth weight in kilograms using Malawi demographic health survey data of 2010 study. Full Bayesian method by integrated nested Laplace approximations (INLA) was used to estimate the model. Second order random walk priors were assigned for mother age and antenatal visits for pregnancy while Gaussian markov random field prior was used for district of the child.

**Results:**

Residual spatial patterns reveal areas in the southern region promoting high birth weight while areas in the central and northern region promote low birth weight. Most fixed effects findings are consistent with the literature. Richest family, normal mother body mass index (BMI), mother over weight (BMI > 25 kg/m^2^), birth order 2–3, mother secondary education and height (≥150 cm) negate low birth weight while weight 45–70 kg promote low birth weight. Birth order category 6+, mother height (≥150 cm) and poor wealth quintile, promote high birth weight, while richer and richest wealth quintiles and education categories: primary, secondary, and higher, and mother overweight (BMI > 25 kg/m^2^) reduce high birth weight. Antenatal visits for pregnancy reduce both low and high birth weight.

**Conclusion:**

Strategies to reduce low and high birth weight should simultaneously address mother education, weight gain during pregnancy and poverty while targeting areas increasing low and high birth weight.

## Background

High birth weight (HBW) defined as new born weight greater than 4.5 kg is an emerging public health issue in developing countries [1]. It is generally accepted that HBW occurs in the range of 3–15% of all pregnancies [2]. As an emerging issue, HBW is under reported in sub-Saharan Africa. In Malawi there is no report to my knowledge regarding the prevalence of HBW. HBW according to research [3–10] carries significant risk to both the mother and the child. A high risk of shoulder dystocia has been found to be related with high birth weight [3–5]. Fetal problems like still birth, Erb’s palsy, infant jaundice, and respiratory distress have been identified to be rampant in high birth weight new born babies than normal weight babies [6]. HBW is also a predictor of infant death [7, 8]. Long term effects of high birth weight have been found to be poor mental performance in childhood and high occurence of overweight later in life [9, 10]. Low birth weight (LBW) according to [11] is defined as weight less than 2.5 kg. LBW is also a public health issue as it brings about prenatal and neonatal deaths. World distribution of LBW shows that sub-Saharan Africa has the second highest prevalence of LBW, pegged at 15% and Malawi part of sub Saharan Africa has 12% prevalence [12].

A number of factors influence high birth weight. HBW is associated with gestational diabetes. Birth order, a previous HBW infant, male fetus, maternal and paternal birth weight, ethnicity, gestational hypertension, preeclampsia, and increased interval between pregnancies are also known to be associated with HBW [13]. Nevertheless, studies indicate that high mother body mass index (BMI) and weight gain during pregnancy are the most important determinants of high birth weight [14, 15].

On the other hand, LBW is primarily caused by uterine growth retardation or preterm delivery or both. Smoking, low maternal education, young and advanced maternal age, single marital status, less weight gain during pregnancy, hypertension, genitourinary tract infection in pregnancy, first births and fewer prenatal consultations are other factors of LBW [16–19]. Furthermore, low family income, history of miscarriage is also associated with LBW [20]. In addition, being exposed to air pollutants is also known to be related with low birth weight [21]. Much research has been conducted on understanding factors for low birth weight and little attention has been paid to derterminants of high birth weight.

In addition, most studies that have looked at factors of LBW and HBW have used mean regression instead of quantile regression [22, 23]. In quantile regression we model the relationship between the covariates and the conditional quantiles of the dependent variable. The methodology supplements mean regression and provides a clearer picture of the underlying relationships of interest that can be especially useful when relationships in the extreme distribution of the response are of interest as it is the case in this study for low or high birth weight relationship with covariates.

The purpose of this research article was to investigate factors of low and high birth weight using the quantile regression so as to have an understanding of effects of covariates on the extreme distribution of the response. The modeling frame work also modeled metrical covariates non parametrically so as to capture their subtle influences. In addition, there was incorporation of spatial random effects so as to take into account the effect of unobserved area level covariates. Incorporation of spatial effects avoided the underestimation of model parameters’ standard errors which if not incorporated could result in erroneous rejection of null hypothesis about significance of the covariates.

The article content is presented as follows: first, methods in terms of study population, area, data and statistical analysis are presented. Results, discussion and conclusion follow there after.

## Methods

### Data and materials

The study looked at children in Malawi less than 5 years and used secondary data (2010 Malawi demographic and health survey (MDHS) data). The data was accessed from the demographic health survey (DHS) website after being given permission. According to [12], the Malawi Health Research Committee, Institutional Review Board of ICF Macro and the Centre for Disease and Control (CDC) in Atlanta, USA gave the ethical approval for the MDHS study. For details on how study was designed refer to [12]. The map file for the spatial effects was provided by the Malawi National Statistical Office (NSO) licensed under Open Government Licence v.3.0 Additional file 1.

Stata version 12 (StataCorp, Texas) was used to extract and generate new variables in the data. Variables found in previous studies on child birth weight were used in this study. Child birth weight in kilograms was the dependent variable in the extracted data set and the independent variables were mother smoking status, mother age, mother education, mother height, mother weight, mother BMI, number of antenatal visits for pregnancy, birth order, wealth index and district of the child. Birth order, mother smoking status, mother education, wealth index, mother height, mother weight, mother BMI and district of the child were categorical variables. The inclusion of mother BMI, weight and height instead of either BMI or the two, weight and height was based on previous research [24] who included all the three variables. In addition, BMI separately was being used as proxy measure of mother nutrition status where low BMI means under nutrition (underweight) and high BMI means overweight, and height in particular was being used as a proxy measure of hereditary factor in influencing LBW and HBW where normally tall mothers tend to give birth to high birth weight babies and vice versa. Mother weight was being used a proxy of mother weight gain during pregnancy as there was no weight gain variable in the DHS data. The total number of live child births was 19,697. Out of this number, 13087 child births had their birth weight reported either by mother recall or from record. The missing covariate values in data set were not removed.

### Statistical analysis

First bivariate quantile regression was performed in R using quantreg package to select potential independent variables for the multiple variable regression. Independent variables that were significant at 20% significance level were taken as candidate variables for multiple variable regression. The 20% significance level was used in selecting independent variables for multiple variable regression, to allow more potential independent variables to be selected. Cross tabulation between factor independent variables and categorized birth weight (less than 2.5 kg, 2.5 kg to 4.5 kg and greater than 4.5 kg) was performed to have percentage distribution of low and high birth weight per factor variable levels.

Multiple variable quantile regression models were then fitted in R using INLA package. Quantile regression models the conditional quantiles on the covariates instead of the mean. Let *y*_*i*_ be the dependent variable and *X*_*i*_ = (*X*_*i*1_, *X*_*i*2_, …, *X*_*ip*_) be the vector of p independent variables. The quintile regression of *y*_*i*_ on *X*_*i*_ is defined as

1 $$ q\left(\tau |{X}_i\right)={X}_i^{\prime }{\gamma}_{\tau } $$

where *p*(*y*_*i*_ < *q*(*τ*| *X*_*i*_)) = *τ ϵ* [0, 1] is the quantile level and *γ*_*τ*_ = (*γ*_1_(*τ*), *γ*_2_(*τ*), …, *γ*_*p*_(*τ*)) is the vector of p covariates effects. The value of *τ* considered in this study was 0.05 and 0.95 where 0.05 corresponds to low birth weight (birth weight = 2.5 kg), and 0.95 corresponds to high birth weight (birth weight = 4.5 kg). If *τ* = 0.5, then we have the usual linear regression, that is, mean regression. Model (1) assumes that the predictor is linear and in the presence of non linear and spatial predictors (1) becomes:

2 $$ q\left(\tau |{X}_i\right)={w}_i^T{\gamma}_{\tau }+{f}_{1\left(\tau \right)}\left({x}_{i1}\right)+{f}_{2\left(\tau \right)}\left({x}_{i2}\right)+\dots +{f}_{p\left(\tau \right)}\left({x}_{ip}\right) $$

where *f*_*j*_ for *j* = 1, 2, 3, …, *p* represent the effect of non linear independent variables including the spatial effect. The vector of coefficients *γ*_*τ*_ determine the parametric relationship between the response and the categorical covariates. The two unknown parameters *γ*_*τ*_, *f*_*j*(*τ*)_ are normally estimated via minimization problem

$$ \left(\begin{array}{c}\mathit{\min}\\ {}{\gamma}_{\tau },{f}_{j\left(\tau \right)}\end{array}\right)=\sum {\rho}_{\tau }{n}_{\tau_i}+{\lambda}_0\mid \left|{\beta}_{\tau}\right|\mid 1+\sum \limits_{j=1}^q{\lambda}_jV\left({\nabla}_{\tau_i}\right) $$

where *ρ*_*τ*_ is the loss function for the given *τ* and *λ*_0_ is the intial smooth paramer for function *f*_*j*(*τ*)_ and *λ*_*j*_ is *j* smooth parameter, $\left|\left|{\beta}_{\tau}\right|\right|1=\sum \limits_{k=1}^k\mid {\beta}_{\tau_k}\mid$ and $V\Big(\left({\nabla}_{\tau_i}\right)$ denote the total variation of the derivative on the gradient of the function ${f}_{\tau_i}$.

Since model inference was fully Bayesian, all parameters were assigned priors. The smooth functions for metrical/spatial covariates *f*_*τ*_(.) were assigned functions from Gaussian markov random field (GMRF) family, that is, if *f*_*τ*_(.) has mean *μ* and precision matrix *δQ*, it is assumed to have the density of the form

$$ \left[f|\delta \right]\propto {\delta}^{n-m/2}\exp \left(-\frac{\delta }{2}{\left(f-\mu \right)}^{\prime }Q\left(f-\mu \right)\right) $$

where *Q* is the semi-definite matrix of constants with rank *n* − *m*(*m* ≥ 0) which also determines the kind of the particular GMRF. For the metrical covariates, we selected the second order random walk (RW2) priors [25], and the spatial effects were assigned the intrinsic conditional autoregressive (ICAR) [26]. The selection of RW2 prior for metrical covariates was to be more flexible in capturing the nonlinear relationship considering that RW2 prior corresponds to the locally quadratic fit and first order random walk prior (RW1) corresponds to locally linear fit according to [27]. The selection of GMRF in general for all non linear terms was from the fact that the use of GMRF prior was appropriate for the spatial covariate as data was based on the level of geographical region (district), that is, the exact data locations were not known so as to use the two dimensional penalized spline according to [27]. In this case if *n*_*i*_ denotes the number of neighbours for site *s*_*i*_ then the spatial effect prior distribution is

$$ f\left({s}_i|{s}_j\right)\sim N\left(\frac{1}{n_i}\sum \limits_{i\ne j}^{n_i}f\left({s}_j\right),\frac{1}{\delta {n}_i}\Big)\right) $$

The fixed effects were assigned diffuse priors i.e. [*γ*_*τ*_] ∝ *constant*. In this case we assumed prior ignorance for the fixed effects and had let the data speak for itself in estimation of parameters.

Inference of joint posterior distribution of model parameters was by integrated nested laplace approximations (INLA). Other methods like markov chain monte carlo (MCMC) could be used to infer about posterior distribution but INLA approach is faster for the quantile regression than MCMC according to [28]. Model inference was not design based, as this is common in Bayesian inference [29]. According to [30] the true Bayesian analyst does not use survey weights as the focus is on reliable statistical models rather than on assessing the degree to which their estimates are nationally representative or not.

## Results

Since the bivariate analysis was similar to that of [22] the results here are almost the same. Young mothers aged 20 years or less and older mothers aged 35–49 years, have higher prevalence of low child birth weight than mothers aged 20–34 years, and prevalence of high birth weight seems to increase with increase in mother age (X^2^ = 24.93, *p* < 0.001) (Table 1). Similar to [22], in terms of birth order number, the prevalence of low birth weight babies is higher for first born babies than babies born later while prevalence of high birth weight is higher for higher order number babies (X^2^ = 28.41, *p* < 0.001). An indirect relationship exists between low or high birth weight and mother education (X^2^ = 19.1, *p* < 0.001). The same relationship is observed between low or high birth weight and wealth quintile (X^2^ = 20.49, *p* < 0.001). In this case, the percentage of low or high birth weight babies decreases with increase in education and household wealth. Looking at the regions (Table 2), central region has the highest prevalence of low birth weight infants and the southern region has the smallest, that is, 14 and 11% respectively. The central and southern regions have relatively higher prevalence of high birth weight (5.7%) than northern region (4%). Districts in the northern region do not vary in proportion of children with low or high birth weight (X^2^ = 6.78, *p* = 0.148) compared to districts in the central (X^2^ = 19.90, *p* = 0.011) and southern region (X^2^ = 26.97, *p* = 0.008).

**Table 1:** Percentage distribution of low and high birth weight for some covariates and the bivariate Pearson Chi-square test

Variable	Birth weight less than 2.5 kg	Birth weight more than 4.5 kg	Pearson Chi-squared (*P*-value)
Mother age at birth			
< 20	15.4	4.7	24.93 (<0.001)
20–34	11.2	5.1	
35–49	14.5	6.6	
Birth order			
1	15.0	4.4	28.41 (<0.001)
2–3	11.0	4.6	
4–5	11.0	5.8	
6+	13.3	7.2	
Mother smoking			
Smoke tobacco	14.0	5.6	0.02 (0.992)
Does not	12.3	5.3	
Mother education			
No education	13.3	8.0	19.10 (<0.001)
Primary	12.8	5.5	
Secondary	10.2	2.8	
Higher	7.0	1.9	
Wealth index			
Poorest	13.5	6.3	20.49 (<0.001)
Poor	13.2	6.7	
Rich	12.6	5.7	
Richer	11.8	4.6	
Richest	10.6	3.1

**Table 2:** Prevalence of low and high birth weight by district and bivariate Pearson Chi-squared test

District	Birth weight less than 2.5 kg	Birth weight more than 4.5 kg	Pearson Chi-square
Northern Region	11.6	4.0	6.78 (0.148)
Chitipa	9.6	3.3	
Karonga	8.9	10.4	
Nkhata-bay	9.6	1.2	
Rumphi	9.5	2.8	
Mzimba	13.6	3.7	
Central Region	13.5	5.7	19.90 (0.011)
Kasungu	11.9	6.5	
Nkhota-kota	11.3	5.3	
Ntchisi	12.3	6.0	
Dowa	13.1	4.8	
Salima	11.0	4.5	
Lilongwe	17.2	4.9	
Mchinji	14.8	3.0	
Dedza	13.0	8.6	
Ntcheu	9.2	7.4	
Southern Region	11.3	5.7	26.97 (0.008)
Mangochi	9.4	6.8	
Machinga	9.5	6.4	
Zomba	10.1	6.6	
Chiradzulu	12.2	8.2	
Blantyre	12.6	5.9	
Mwanza	9.3	4.0	
Thyolo	16.7	4.5	
Mulanje	11.1	3.7	
Phalombe	9.9	9.5	
Chikhwawa	10.5	4.3	
Nsanje	7.5	5.3	
Balaka	11.0	3.8	
Neno	16.9	4.2	

Table 3 presents results from the fitted quantile regression models of birth weight at 5, and 95% percentile. The 5% percentile corresponds to low birth weight (birth weight < =2.5 kg) and 95% percentile corresponds to high birth weight (birth weight= > 4.5 kg). The model fit statistics (deviance information criterion (DIC) and the marginal log-likelihood (LL)) show that the model at 5% percentile fit the data well as it has the smaller DIC and larger log-likelihood than model at 95%. The fixed effect variables significant to low birth weight with positive association are wealth category of richest family (coefficient: 0.1365; 95% CI: 0.0807, 0.1923), smoking (coefficient: 0.2409; 95% CI: 0.0708, 0.4100), mother normal BMI (coefficient: 0.3587; 95% CI: 0.2609, 0.4569), mother BMI > 25 kg/m^2^(coefficient: 0.2008; 95% CI: 0.0976, 0.2895), birth order 4–5 (coefficient: 0.0978; 95% CI: 0.0195, 0.1859), mother secondary education (coefficient: 0.1388; 95% CI: 0.0603, 0.2259), mother height ≥ 150 cm (coefficient: 0.2662; 95% CI: 0.1627, 0.3517), and variable with negative association is weight 45–70 kg (coefficient: -0.1559; 95% CI:-0.2627, − 0.0048). For the 95% percentile model, birth order category 6 + (coefficient: 0.1731; 95% CI: 0.0489, 0.3082) and mother height ≥ 150 cm (coefficient: 0.3184; 95% CI: 0.1069, 0.4814) have a positive association with birth weight, that is, they promote high birth weight, while poor wealth index (coefficient: 0.0746; 95% CI: 0.0106, 0.1378), richer wealth index (coefficient: -0.0732; 95% CI: − 0.1378, − 0.0112), richest wealth index (coefficient: -0.1102, 95% CI: − 0.1875, − 0.0335) and education categories: primary (coefficient: -0.2115; 95% CI:-0.2912, − 0.1404), secondary (coefficient: -0.5371; 95% CI:-0.6266, − 0.4518), higher (coefficient: -0.8267; 95% CI: − 1.0646, − 0.4284), mother BMI > 25 kg/m^2^(coefficient: -0.2378; 95% CI: − 0.3781, − 0.0697)) are negatively related with birth weight on higher values of birth weight.

**Table 3:** Summary of quantile regression models

Variable	τ = 0.05 (LBW)	τ = 0.95 (HBW)
Normal BMI (18.50–25 kg/m^2^)	0.3587 (0.2609, 0.4569)	−0.0069 (−0.1569, 0.1274)
Mother BMI >25 kg/m^2^	0.2008 (0.0976, 0.2895)	−0.2378 (−0.3781, −0.0697)
Smoke (yes)	0.2409 (0.0708, 0.4100)	0.3155 (−0.0044, 0.6142)
Birth order 2–3	− 0.0165 (− 0.0688, 0.0363)	0.0155 (− 0.0513, 0.0858)
Birth order 4–5	0.0978 (0.0195, 0.1859)	0.0793 (− 0.0152, 0.1772)
Birth order 6+	− 0.0120 (− 0.1163, 0.1034)	0.1731 (0.0489, 0.3082)
Primary education	0.0213 (− 0.0346, 0.0838)	− 0.2115 (− 0.2912, − 0.1404)
Secondary education	0.1388 (0.0603, 0.2259)	− 0.5371 (− 0.6266, − 0.4518)
Higher education	0.0511 (− 0.3984, 0.3367)	−0.8267 (−1.0646, − 0.4284)
Poor	−0.0141 (− 0.0706, 0.0530)	0.0746 (0.0106, 0.1378)
Rich	0.0364 (−0.0208, 0.0942)	0.0479 (− 0.0167, 0.1105)
Richer	−0.0128 (− 0.0694, 0.0500)	−0.0732 (− 0.1378, − 0.0112)
Richest	0.1365 (0.0807, 0.1923)	−0.1102 (−0.1875, − 0.0335)
Mother height ≥ 150 cm	0.2662 (0.1627, 0.3517)	0.3184 (0.1069, 0.4814)
Mother weight (45-70 kg)	−0.1559 (−0.2627, 0.0048)	−0.1777 (−0.4115, 0.0417)
Mother weight > 70 kg	0.0680 (−0.0438, 0.2173)	−0.1757 (− 0.4389 0.0197)
Variance parameters		
Mother age	0.1412	0.2004
Antenatal visits	0.0165	0.0001
Structured spatial effects	0.0121	0.0014
Model fit statistics		
LL	−19,390.79	−22,565.51
DIC	38,357.7	44,731.5

**Fig. 1:**
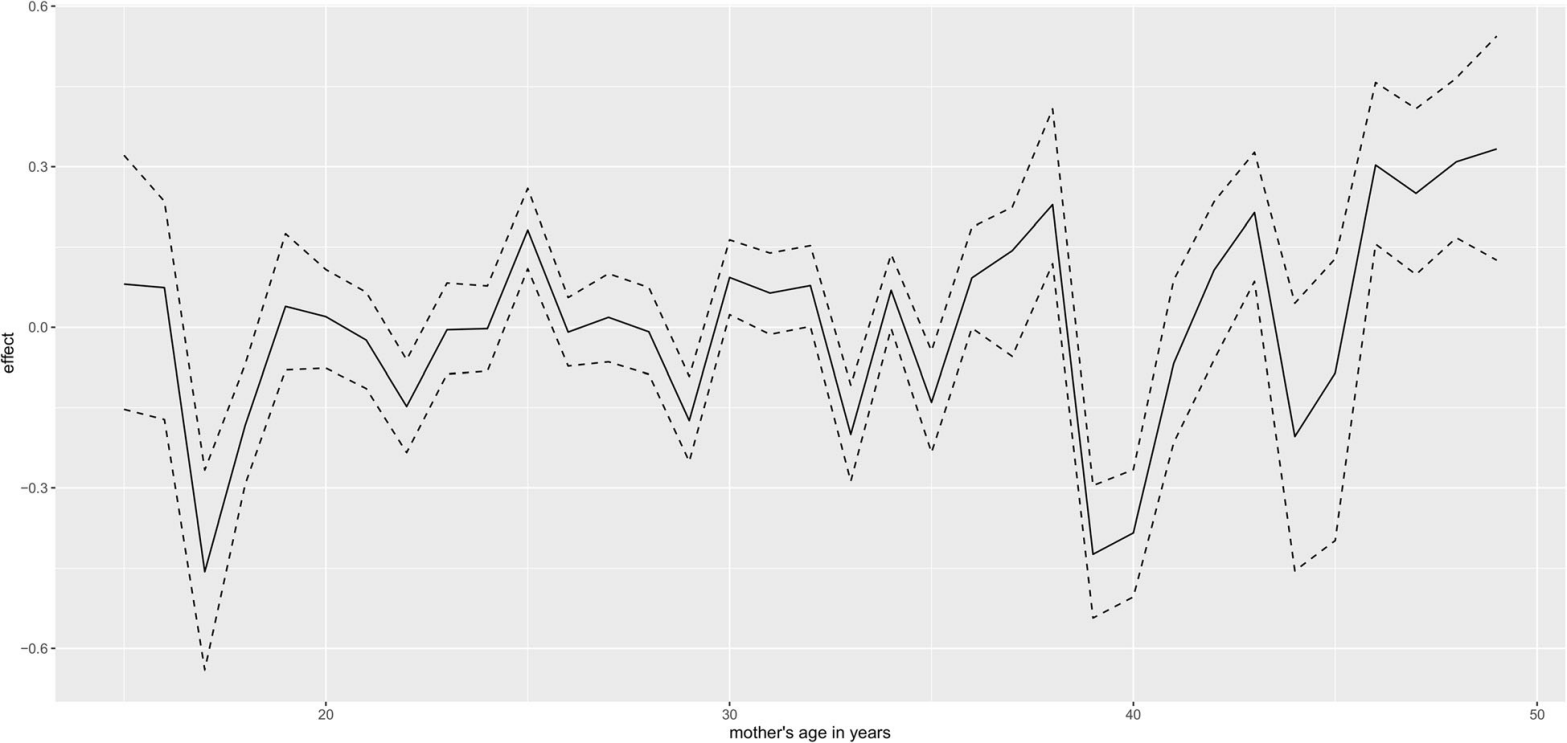
Non linear effect of mother age to low birth weight

**Fig. 2:**
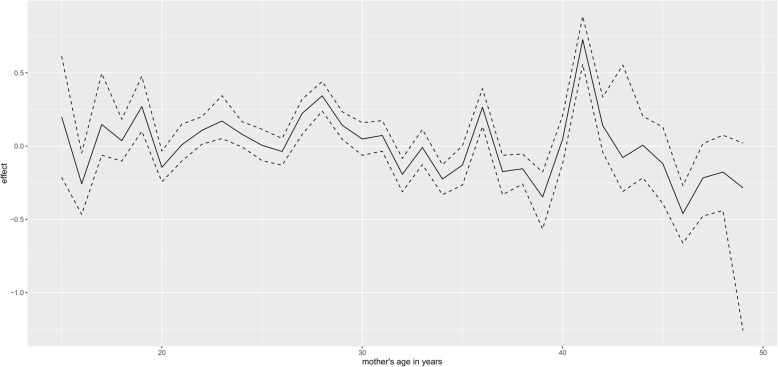
Non linear effect of mother age to high birth weight

**Fig. 3:**
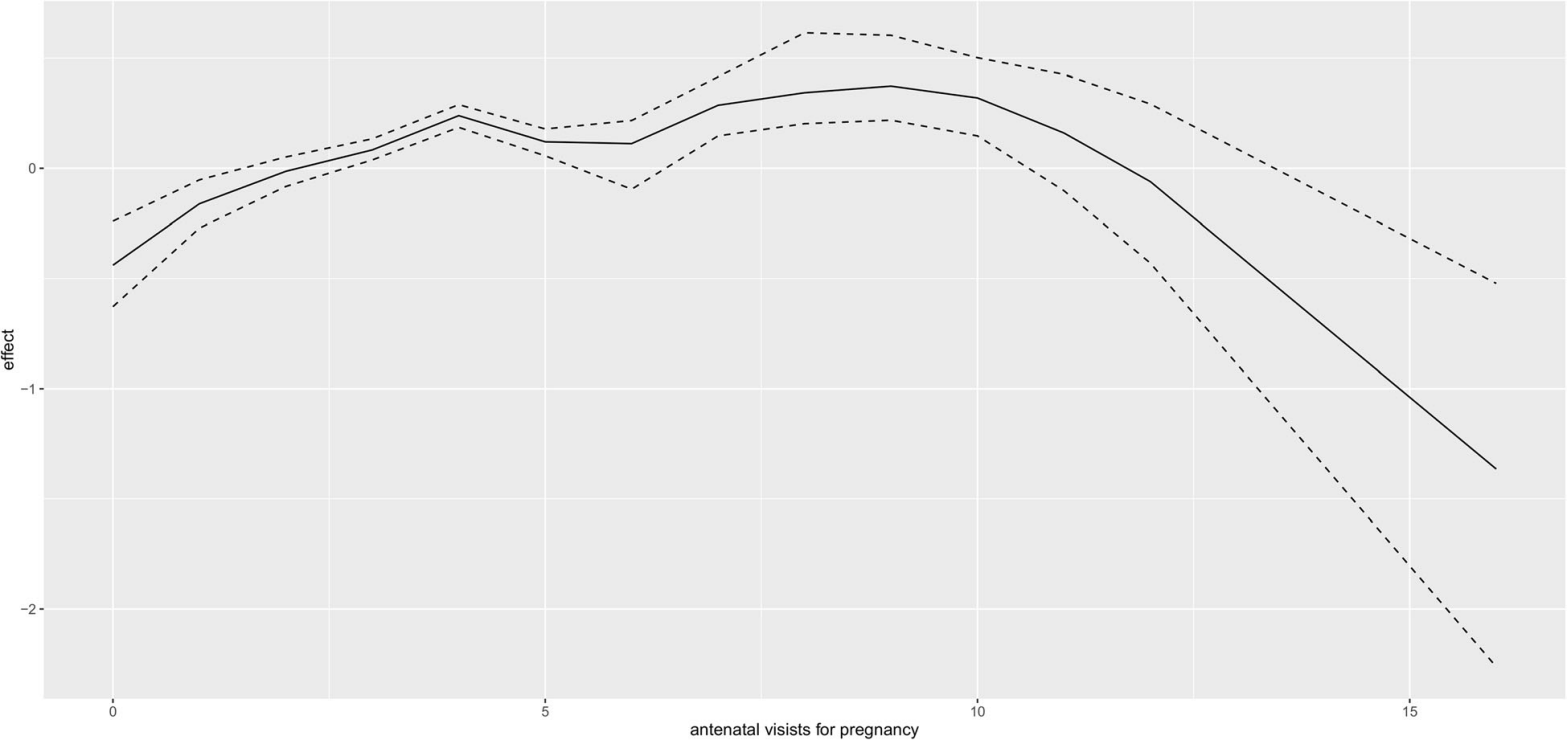
Non linear effect of antenatal visits to low birth weight

**Fig. 4:**
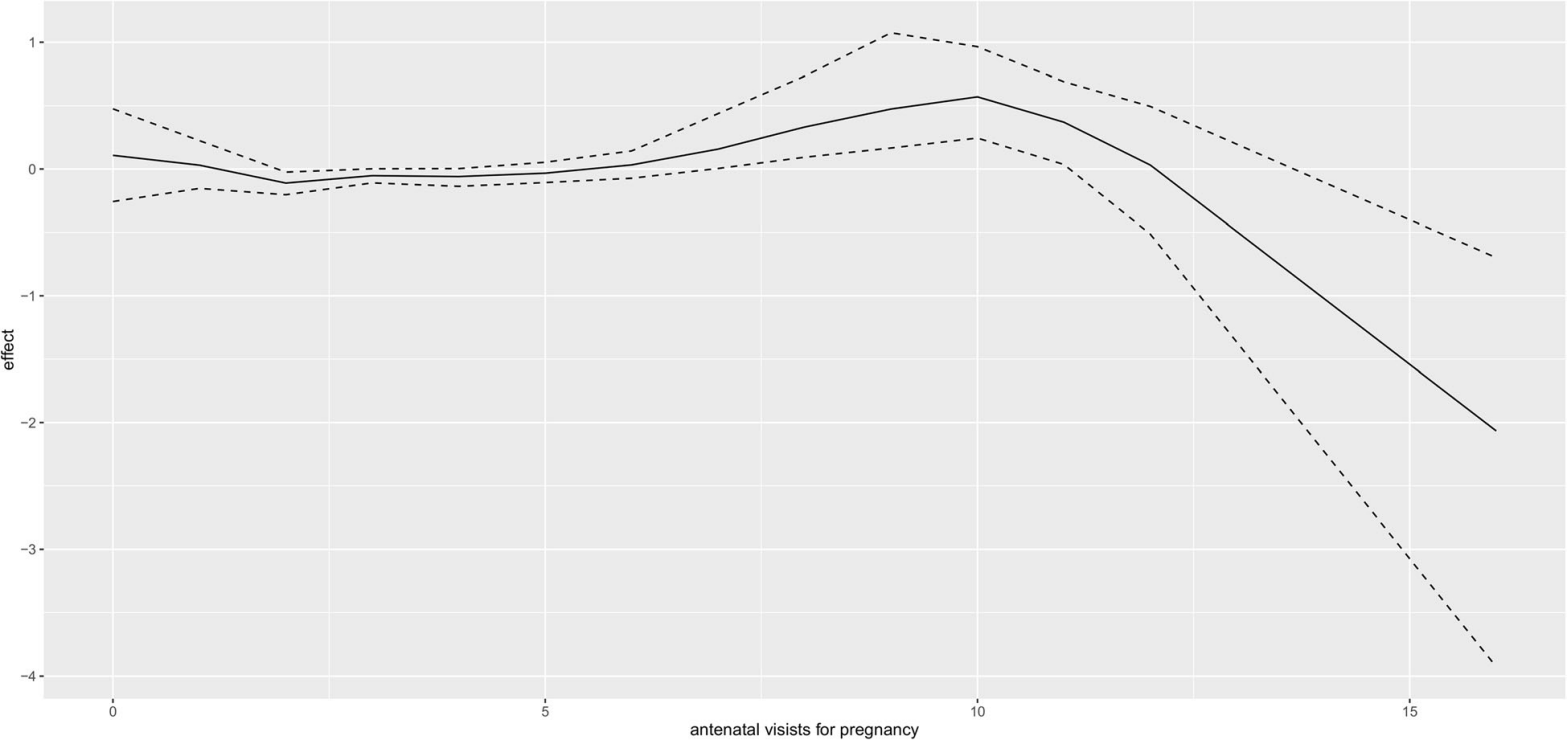
Non linear effect of antenatal visits to high birth weight

Figure 1 presents non linear effect of mother age to low birth weight. There seems almost constant effect of mother age in small fluctuating amplitude manner on child low birth weight, that is, as mother age increases, its effect on child birth weight remains more or less the same. The same trend is observed for the effect of mother age on high birth weight (Fig. 2). For the non linear effect of mother antenatal visits for pregnancy to low birth weight (Fig. 3), as the number of antenatal visits increases, birth weight also increases but then, as it further increases, birth weight start to drop. The same effect trend is observed for the effect of antenatal visits on high birth weight (Fig. 4) with a weaker positive effect for the fewer antenatal visits.

**Fig. 5:**
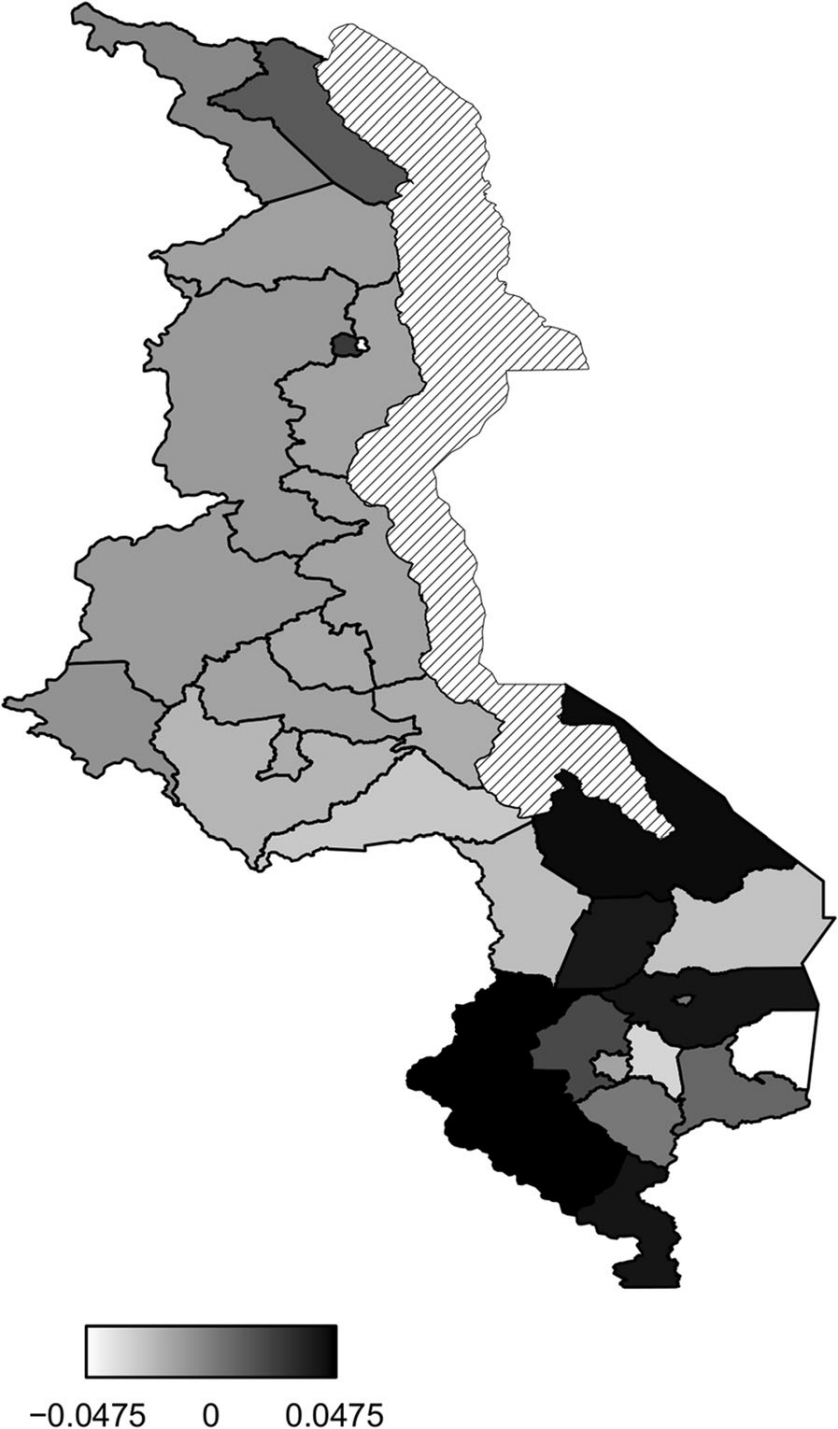
Structured spatial effect to low birth weight (Map file source: National Statistical Office (www.nsomalawi.mw) licensed under the Open Government License v.3.0)

**Fig. 6:**
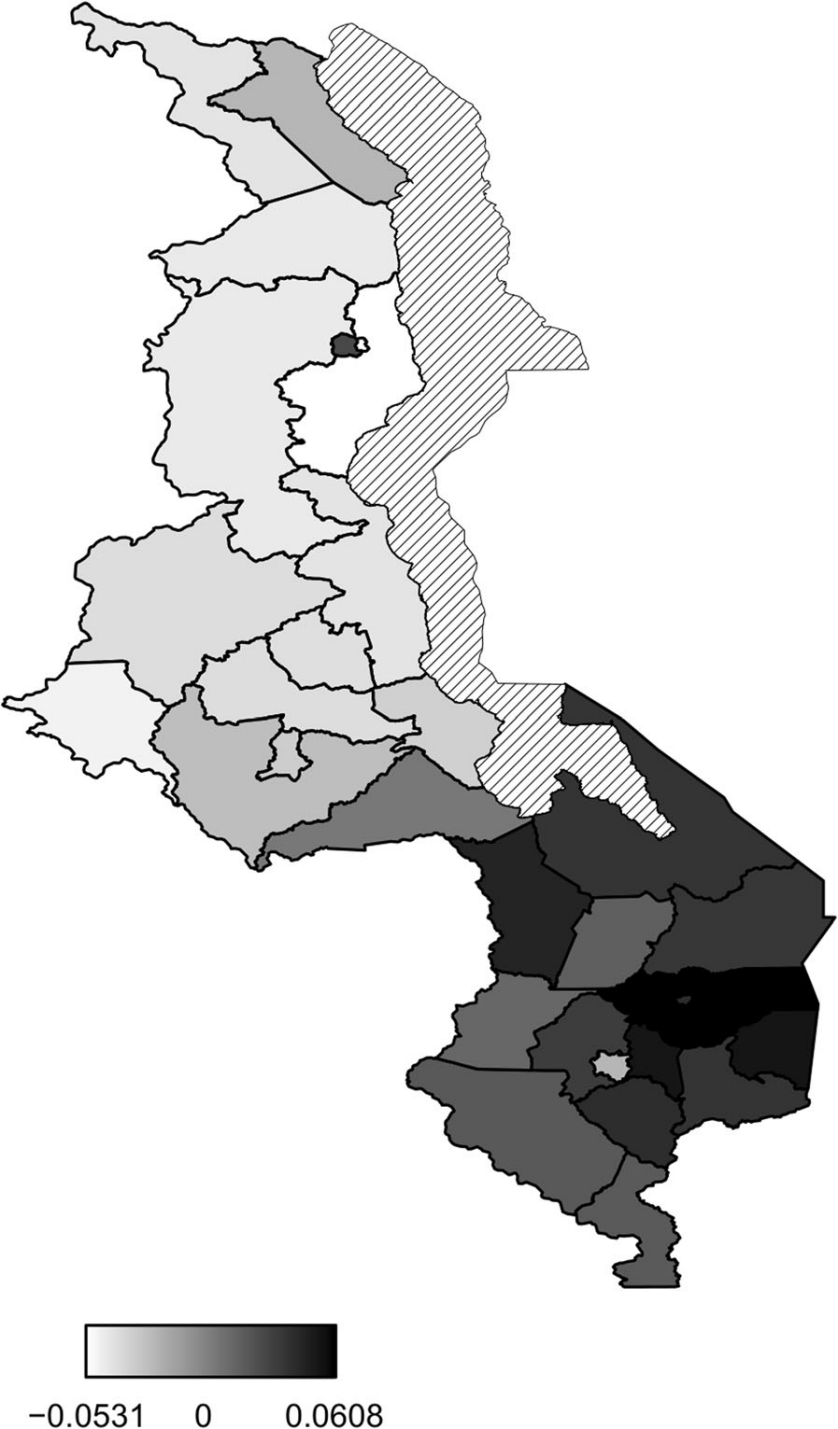
Structured spatial effect to high birth weight (Map file source: National Statistical Office (www.nsomalawi.mw) licensed under the Open Government License v.3.0)

**Fig. 7:**
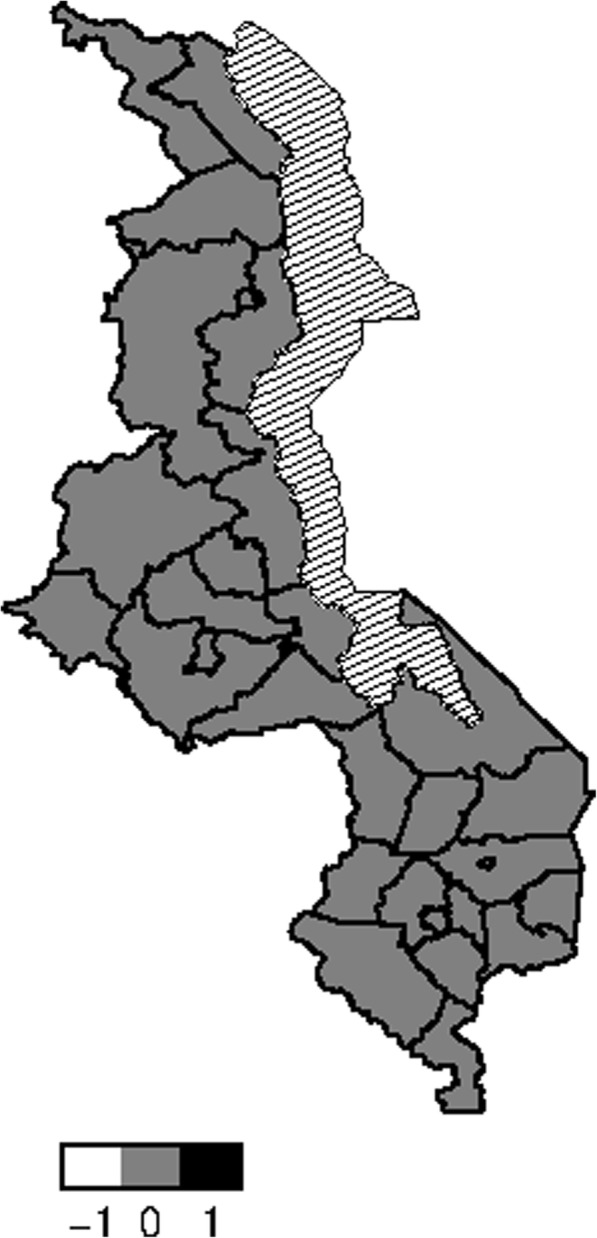
95% credible intervals map of spatial effect to LBW (white means negative effect, grey means insignificant, black means positive effect, and white with lines means lake) (Map file source: National Statistical Office (www.nsomalawi.mw) licensed under the Open Government License v.3.0)

**Fig. 8:**
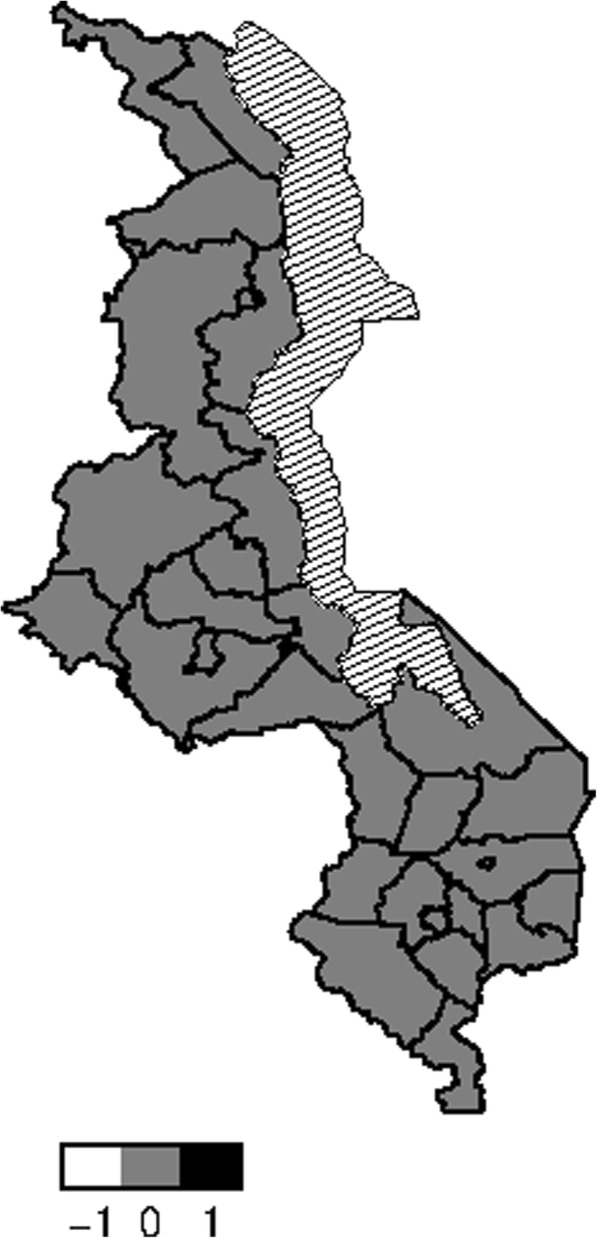
95% credible intervals map of spatial effect to HBW (white means negative effect, grey color means insignificant, black color means positive effect, and white with lines means lake) (Map file source: National Statistical Office (www.nsomalawi.mw) licensed under the Open Government License v.3.0)

Figure 5 presents structured spatial effects to low birth weight. Spatial effects are the surrogates of correlated unobserved influences to the response (e.g birth weight). There seems residual spatial variation to childhood low birth weight with the most areas in the south inhibiting low birth weight where as areas in the central and northern region promote low birth weight. The same pattern is observed for the structured spatial effect on higher values of birth weight (Fig. 6), that is, areas in the south promote high birth weight while areas in centre and north inhibit high birth weight. Both spatial effects were not significant though as the credible intervals maps show no color variation among all the districts (Fig. 7 and Fig. 8), that is they all have the same grey color which corresponds to zero, meaning insignificant. For orientation on interpreting posterior probability maps one can read [31, 32].

## Discussion

This study employs the use of spatial quantile regression to explore the relationship of birth weight with its covariates at different quantiles of birth weight while taking into account residual spatial effects. Investigating covariate effect at different response quartiles is important as relationship may be different at different levels of the response. Not only relationships may be different at different quantiles of the response but also the significance of these relationships. For example in this study, birth order category 4–5 is significant at 5% percentile and is insignificant at 95% percentile. Quantile regression also allows one to model percentiles of interests of the data, for example, extreme values of the data, as it is the case in this study where the interest was to investigate factors affecting high and low birth weight.

Fixed effects factors found as significant predictors of lower values of birth weight is wealth category of richest family, smoking status, mother normal BMI, mother over weight (BMI > 25 kg/m^2^), birth order category 2–3, mother secondary education, and height (≥150 cm) with positive association and variables with negative association (promoting low birth weight) is weight 45-70 kg. For the 95% percentile model, birth order category 6+ and mother height (≥150 cm) and poor wealth quintile have a positive association with birth weight, that is, they promote high birth weight, while richer wealth index, richest wealth quintile and education categories: primary, secondary, and higher, mother overweight (BMI > 25 kg/m^2^) have a negative association with birth weight. Poor wealth quantile promoting high birth weight may seem unexpected but sometimes it has been found like this, for example [33] found a similar result where low socio economic status was associated with high birth weight in British Colombia region. The possible cause would be that, normally poverty is associated with improper diet, for example eating fats only which would in turn increase birth weight. Birth order positive association with birth weight is in agreement with [34] where birth order was found as an important factor influencing birth weight and that first order babies are more likely to be small babies than higher order births. Family wealth index of richest category is positively correlated with low birth weight and negatively correlated with high birth weight. According to [1], “family wealth index is a proxy measure of socio economic status of the child and high socio-economic status people are better able to avoid negative health outcomes and therefore will have a lower incidence of high birth weight”, at the same time will not have low birth weight. Mother body mass index behaves in the similar manner as family wealth by being negatively correlated with high birth weight and positively correlated with low birth weight. Smoking positive correlation with birth weight across all the two modeled quantiles contradicts the common literature finding [3, 35], though its effect is not significant at 95% percentile. The unexpected effect of smoking on birth weight is likely due to lack of taking into account unmeasured positive confounders of smoking on birth weight. Future research therefore can carefully include all possible confounders of smoking to accurately investigate its effect on birth weight. Education relationship with low and high birth weight values is positive and negative respectively, implying negating both extreme values. This is so as mothers who are educated are likely to eat proper diet to reduce low birth weight but also they may also know the dangers of over nutrition unlike uneducated mothers there by avoiding high birth weight children. Mother height has positive relationship with birth weight on lower and higher values of birth weight. This relationship is making sense since taller children will inherit their taller mothers and hence larger birth weight as height is directly proportional to weight. Mother higher weight during pregnancy (weight > 70 kg) promote high birth weight for lower birth weight values and at same time reduce high birth weight for high birth weight values though not significant at these extreme values, that is, it negate both extreme birth weight outcomes (low and high).

The nonlinear effect of mother age on birth weight is almost the same on lower and high values of birth. With prenatal visits, there is a positive association with low values of birth weight for the fewer number of antenatal visits and a negative correlation with increased number of antenatal visits. For high birth weight, the effect of antenatal visits start in flattening out mode and then ends with strong reducing effect. This finding is consistent with [36] where antenatal visits had a stronger increasing effect for lower values (negating low birth weight) but then flattens out for larger values.

The residual spatial patterns observed in low and high child birth weight are likely due to unmeasured factors not represented in the models, and it is a matter of suggestion to find them. According to [37], the area natural resources such as soil type and land slope, area population density, and distance to health facilities would be some of these factors. In this case, things like soil type and slope may have an impact on crop yield which may have an effect on mother nutrition. Number of people per unit area may affect spatial distribution in child birth weight in the way that, high number of people per unit area may induce competition for food in the area which may affect mother nutrition. Maternal nutrition in turn may directly affect child birth weight. Time taken to go to health facility affect mother number of times of going to the health facilities for prenatal care which can have an impact on baby birth weight. The spatial disparities observed between the south, and both centre and north could be from the fact that the southern region is relatively more developed than the north and centre in terms of hospitals, roads and schools among others and hence more likely to have improved maternal health indicators like nutrition which have an impact on child birth weight. For example according to MDHS 2010 report [12] on mother micro nutrient intake, women in the southern region have relatively more days of receiving iron tablet or syrup during their pregnancy than their counterparts in the northern and central region.

The study did not go without weaknesses. Since the study was cross sectional in design, no causal inference can be made between the outcome and the independent variables. In addition, since the study used secondary data, there was limitation in the inclusion of some variables, that is, variables not in the data but known to be associated with outcome. For instance, pregnancy history which is known to influence birth weight according to [38] could not be incorporated in the study.

## Conclusion

The study supports non linear modeling of some covariates like mother age and antenatal visits that show non-linear effect. Nonetheless there is no support of incorporating location as a spatial effect as there was no significant spatial variation of child birth weight. Nonetheless, the spatial patterns shown reveal the effect of unmeasured variables with some spatial dependence or may be epidemiological processes that are responsible for this spatial dependence, and the maps created can be used for prioritizing the areas with high risk. Strategies to minimize low/high birth weight must include education for mothers, weight gain during pregnancy in this study approximated by mother weight, and poverty reduction especially in areas shown to increase low/high birth weight.

## Supplementary information

**Additional file 1.** Child birth weight data.

## Data Availability

All data generated or analyzed during this study are included in this published article [and it is additional file].

## Abbreviations

BMI: Body mass index
CDC: Centre for disease and control
DIC: Deviance information criterion
GMRF: Gaussian markov random field
HBW: High birth weight
ICAR: Intrinsic conditional autoregressive
INLA: Integrated nested laplace approximations
LBW: Low birth weight
MCMC: Markov chain monte carlo
MDHS: Malawi demographic health survey
NSO: National statistical office
